# Supplementary material for: Phenotypic and Genotypic screening of fifty-two rice (Oryza sativa L.) genotypes for desirable cultivars against blast disease
Source: PLoS One. 2023 Mar 10;18(3):e0280762. doi: 10.1371/journal.pone.0280762 (PMC10004593; doi:10.1371/journal.pone.0280762)
Supplement: Attachment — Submitted filename: PONE-D-22-18593 (1).pdf [file pone.0280762.s001.pdf]

PLOS ONE

Phenotypic and Genotypic screening of fifty-two rice (Oryza sativa L.) germplasms for desirable cultivars against blast disease

--Manuscript Draft--

**Manuscript Number:**	PONE-D-22-18593
**Article Type:**	Research Article
**Full Title:**	Phenotypic and Genotypic screening of fifty-two rice (Oryza sativa L.) germplasms for desirable cultivars against blast disease
**Short Title:**	Screening of desirable rice cultivars against blast disease
**Corresponding Author:**	Akbar Hossain, Ph.D<br>Bangladesh Wheat and Maize Research Institute (BWMRI)<br>Dinajpur, Rangpur Division BANGLADESH
**Keywords:**	Magnaporthe oryzae; Rice blast; Phenotyping; Resistance genes; Molecular markers
**Abstract:**	Magnaporthe oryzae , the rice blast fungus, is one of the most dangerous rice pathogens, causing considerable crop losses around the world. In order to explore the rice blast-resistant sources, ~~we initially performed a~~ A large-scale screening of 277 rice accessions was done. In parallel with field evaluations, fifty-two rice accessions were genotypedfor 25 major blast resistance genes utilizing functional/gene-based markers based on their reactivity against rice blast disease. According to the phenotypic examination, 29(58%) and 22 (42%) entries were found to be highly resistant, 18 (36%) and 29 (57%)<br>showed moderate resistance, and 05 (6%) and 01 (1%), respectively, were highly susceptible to leaf and neck blast. The genetic frequency of 25 major blast resistance genes ranged from 32 to 60%, with two genotypes having a maximum of 16 R -genes each. The 52 rice accessions were divided into two groups based on cluster and population structure analysis. The highly resistant and moderately resistant accessions are divided into different groups using the principal coordinate analysis. According to the analysis of molecular variance, the maximum diversity was found within the population, while the minimum diversity was found between the populations. Two markers (RM5647 and K39512), which correspond to the blast-resistant genes Pi36 and Pik , respectively, showed a significant association to the neck blast disease, whereas three markers (Pi2-i, Pita3, and k2167), which correspond to the blast-resistant genes Pi2 , Pita/Pita2 , and Pikm , respectively, showed a significant association to the leaf blast disease. The associated R-genes might be utilized in rice breeding programmes through marker-assisted breeding, and the identified resistant rice accessions could be used as prospective donors for the production of new resistant varieties in India and around the world.
**Order of Authors:**	Jeevan B., Ph.D
	Rajashekara Hosahatti
	Prasanna S. Koti
	Vinaykumar Hargi Devappa
	Umakanta Ngangkham
	Pramesh Devanna
	Manoj Kumar Yadav
	Krishna Kant Mishra
	Jay Prakash Aditya
	Palanna Kaki Boraiah
	Ahmed Gaber
	Akbar Hossain, Ph.D
**Additional Information:**	

Powered by Editorial Manager® and ProduXion Manager® from Aries Systems Corporation

Question	Response
**Financial Disclosure**<br><br>Enter a financial disclosure statement that describes the sources of funding for the work included in this submission. Review the submission guidelines for detailed requirements. View published research articles from *PLOS ONE* for specific examples.<br><br>This statement is required for submission and **will appear in the published article** if the submission is accepted. Please make sure it is accurate.<br><br>**Unfunded studies**<br>Enter: *The author(s) received no specific funding for this work.*<br><br>**Funded studies**<br>Enter a statement with the following details:<br>• Initials of the authors who received each award<br>• Grant numbers awarded to each author<br>• The full name of each funder<br>• URL of each funder website<br>• Did the sponsors or funders play any role in the study design, data collection and analysis, decision to publish, or preparation of the manuscript?<br>• **NO** - Include this sentence at the end of your statement: *The funders had no role in study design, data collection and analysis, decision to publish, or preparation of the manuscript.*<br>• **YES** - Specify the role(s) played.<br><br>* typeset	The funders had no role in study design, data collection and analysis, decision to publish, or preparation of the manuscript.
**Competing Interests**<br><br>Use the instructions below to enter a competing interest statement for this submission. On behalf of all authors, disclose any competing interests that could be perceived to bias this work—acknowledging all financial supportand any other relevant financial or non-financial competing interests.	The authors have declared that no competing interests exist

Powered by Editorial Manager® and ProduXion Manager® from Aries Systems Corporation

This statement is required for submission and **will appear in the published article** if the submission is accepted. Please make sure it is accurate and that any funding sources listed in your Funding Information later in the submission form are also declared in your Financial Disclosure statement.<br><br>View published research articles from *PLOS ONE* for specific examples.<br><br>**NO authors have competing interests**<br><br>Enter: *The authors have declared that no competing interests exist.*<br><br>**Authors with competing interests**<br><br>Enter competing interest details beginning with this statement:<br><br>*I have read the journal's policy and the authors of this manuscript have the following competing interests: [insert competing interests here]*<br><br>* typeset	
**Ethics Statement**<br><br>Enter an ethics statement for this submission. This statement is required if the study involved:<br><br>• Human participants<br>• Human specimens or tissue<br>• Vertebrate animals or cephalopods<br>• Vertebrate embryos or tissues<br>• Field research<br><br>Write "N/A" if the submission does not require an ethics statement.<br><br>General guidance is provided below. Consult the submission guidelines for detailed instructions. **Make sure that all information entered here is included in the Methods section of the manuscript.**	N/A

Powered by Editorial Manager® and ProduXion Manager® from Aries Systems Corporation

**Format for specific study types**<br><br>**Human Subject Research (involving human participants and/or tissue)**<br>• Give the name of the institutional review board or ethics committee that approved the study<br>• Include the approval number and/or a statement indicating approval of this research<br>• Indicate the form of consent obtained (written/oral) or the reason that consent was not obtained (e.g. the data were analyzed anonymously)<br><br>**Animal Research (involving vertebrate animals, embryos or tissues)**<br>• Provide the name of the Institutional Animal Care and Use Committee (IACUC) or other relevant ethics board that reviewed the study protocol, and indicate whether they approved this research or granted a formal waiver of ethical approval<br>• Include an approval number if one was obtained<br>• If the study involved *non-human primates*, add *additional details* about animal welfare and steps taken to ameliorate suffering<br>• If anesthesia, euthanasia, or any kind of animal sacrifice is part of the study, include briefly which substances and/or methods were applied<br><br>**Field Research**<br><br>Include the following details if this study involves the collection of plant, animal, or other materials from a natural setting:<br>• Field permit number<br>• Name of the institution or relevant body that granted permission	
**Data Availability**<br><br>Authors are required to make all data underlying the findings described fully available, without restriction, and from the time of publication. PLOS allows rare exceptions to address legal and ethical concerns. See the PLOS Data Policy and FAQ for detailed information.	Yes - all data are fully available without restriction

Powered by Editorial Manager® and ProduXion Manager® from Aries Systems Corporation

A Data Availability Statement describing where the data can be found is required at submission. Your answers to this question constitute the Data Availability Statement and **will be published in the article**, if accepted.<br><br>**Important:** Stating 'data available on request from the author' is not sufficient. If your data are only available upon request, select 'No' for the first question and explain your exceptional situation in the text box.<br><br>Do the authors confirm that all data underlying the findings described in their manuscript are fully available without restriction?	
**Describe where the data may be found in full sentences. If you are copying our sample text, replace any instances of XXX with the appropriate details.**<br><br>• If the data are **held or will be held in a public repository**, include URLs, accession numbers or DOIs. If this information will only be available after acceptance, indicate this by ticking the box below. For example: *All XXX files are available from the XXX database (accession number(s) XXX, XXX.*).<br>• If the data are all contained **within the manuscript and/or Supporting Information files**, enter the following: *All relevant data are within the manuscript and its Supporting Information files.*<br>• If neither of these applies but you are able to provide **details of access elsewhere**, with or without limitations, please do so. For example:<br><br>*Data cannot be shared publicly because of [XXX]. Data are available from the XXX Institutional Data Access / Ethics Committee (contact via XXX) for researchers who meet the criteria for access to confidential data.*<br><br>*The data underlying the results presented in the study are available*	All relevant data are within the manuscript

from (include the name of the third party and contact information or URL).<br>• This text is appropriate if the data are owned by a third party and authors do not have permission to share the data.<br><br>* typeset	
Additional data availability information:	

Powered by Editorial Manager® and ProduXion Manager® from Aries Systems Corporation

Manuscript

Click here to access/download;Manuscript;Rice_Manuscript_PLOS ONE.docx

Phenotypic and Genotypic screening of fifty-two rice (*Oryza sativa* L.) germplasms for desirable cultivars against blast disease

Jeevan B. [1,*], Rajashekara Hosahatti [1], Prasanna S. Koti [2], Vinaykumar Hargi Devappa[3], Umakanta Ngangkham [4], Pramesh Devanna [5], Manoj Kumar Yadav [6], Krishna Kant Mishra [1], Jay Prakash Aditya [1], Palanna Kaki Boraiah [3], Ahmed Gaber [7] and Akbar Hossain [8,*]

[1] ICAR-Vivekananda Parvatiya Krishi Anusandhan Sansthan, Almora, Uttarakhand 263601, India; jeevan.bscag@gmail.com (J.B.); rajaiaripath@gmail.com (R.H.); mishrakkpatho@gmail.com (K.K.M.); jayprakashaditya@gmail.com (J.P.A.)

[2] The University of Trans-Disciplinary Health Sciences and Technology, Jarakabande Kaval, Bengaluru, Karnataka 560064, India; prasanskoti@googlemail.com

[3] Project Coordinating Unit, ICAR-AICRP on Small Millets, UAS, GKVK, Bengaluru, Karnataka 560064, India; hargi.vinay@gmail.com (V.H.D.); kbpalanna@gmail.com (P.K.B.)

[4] ICAR- Research Complex for North- Eastern Hill Region, Manipur centre, Imphal, Manipur 795004, India; ukbiotech@gmail.com

[5] Rice Pathology Laboratory, AICRIP, Gangavathi, University of Agricultural Sciences, UAS Campus, Lingsugur Road, Raichur-584 104, Karnataka, India; parmi.iari@gmail.com

[6] Regional Station, ICAR-Indian Agricultural Research Institute, Karnal, Haryana 132001, India; m.yadav14@gmail.com

[8] Department of Biology, College of Science, Taif University, P.O. Box 11099, Taif 21944, Saudi Arabia; a.gaber@tu.edu.sa

[9] Department of Agronomy, Bangladesh Wheat and Maize Research Institute, Dinajpur 5200, Bangladesh

***** All correspondence: jeevan.bscag@gmail.com (J.B.); akbar.hossain@bwmri.gov.bd (A.H.)

Phenotypic and Genotypic screening of fifty-two rice (*Oryza sativa* L.) germplasms for desirable cultivars against blast disease

Abstract

Magnaporthe oryzae, the rice blast fungus, is one of the most dangerous rice pathogens, causing considerable crop losses around the world. In order to explore the rice blast-resistant sources, ~~we~~ initially performed a large-scale screening of 277 rice accessions. In parallel with field evaluations, fifty-two rice accessions were genotyped for 25 major blast resistance genes utilizing functional/gene-based markers based on their reactivity against rice blast disease. According to the phenotypic examination, 29 (58%) and 22 (42 %) entries were found to be highly resistant, 18 (36%) and 29 (57%) showed moderate resistance, and 05 (6%) and 01 (1%), respectively, were highly susceptible to leaf and neck blast. The genetic frequency of 25 major blast resistance genes ranged from 32 to 60%, with two genotypes having a maximum of 16 *R*-genes each. The 52 rice accessions were divided into two groups based on cluster and population structure analysis. The highly resistant and moderately resistant accessions are divided into different groups using the principal coordinate analysis. According to the analysis of molecular variance, the maximum diversity was found within the population, while the minimum diversity was found between the populations. Two markers (RM5647 and K39512), which correspond to the blast-resistant genes *Pi36* and *Pik*, respectively, showed a significant association to the neck blast disease, whereas three markers (Pi2-i, Pita3, and k2167), which correspond to the blast-resistant genes *Pi2*, *Pita/Pita2*, and *Pikm*, respectively, showed a significant association to the leaf blast disease. The associated R-genes might be utilized in rice breeding programmes through marker-assisted breeding, and the identified resistant rice accessions could be used as prospective donors for the production of new resistant varieties in India and around the world.

Keywords: *Magnaporthe oryzae*; Rice blast; Phenotyping; Resistance genes; Molecular markers

1. Introduction

Rice blast disease, caused by filamentous fungus *Magnaporthe oryzae* (anamorph *Pyricularia oryzae*), remains a potential threat to global rice production [1,2]. The blast pathogen can be found in all stages of plants growth and development, causing damage to leaves (leaf blast), nodes (nodal blast), and panicles (neck blast), as well as decreasing grain yield by up to 90% in favourable environmental conditions [3,4,54].

The *M. oryzae* has been documented all over the world and can infect more than 50 host species in the family Poaceae, including rice, wheat, pearl millet, foxtail millet, and finger millet [6 - 8]. Across most of the world's rice-growing regions, including India, blast disease epidemics have occurred [9,10]. Between 1980 and 1987, India experienced several deadly blast disease epidemics in Himachal Pradesh, Tamil Nadu, Andhra Pradesh, and Haryana [11,12].

Chemical fungicides have been useful in controlling the disease, but they are expensive [13,14], ineffective when disease pressure is high [15], and may contribute to pathogen resistance [16]. As a result, the most cost-effective and environmentally acceptable strategy for controlling rice blast disease is to leverage host resistance (*R* genes). Around 118 *R* genes have been discovered so far, with 35 of them being successfully cloned and characterized for leaf blast resistance [17,18]. However, the cloned *R* genes that possess broad-spectrum resistance to leaf blast, have not been tested for neck blast disease [19]. Even though neck blast is the most devastating stage of the disease, there is relatively little information on the genetic processes that underpin neck blast resistance. Nevertheless, 14 QTLs [18] and a few *R* genes have been found for neck blast resistance, including *Pi25(t)* [20], *Pb1* [21], *Pi64* [22], *Pi-jnw1* [23], and *Pi68(t)* [24]. A large majority of the cloned blast *R* genes share nucleotide-binding site (NBS) and leucine-rich repeat (LRR) domains in their protein sequences, except for a few (*Pid2*, *pi21*, and *Ptr*) [17,25,26,]. According to gene-for-gene theory, these *R* genes are race-specific and related to the hypersensitive response (HR) [27]. The *M. oryzae*'s genome contains numerous repetitive DNA and retro-transposons [28], which might cause mutations in genes that mediate the pathogen's virulence and host range, [29 - 31], allowing the fungus to develop new deadly races. The emergence of these races results in a change in pathogenicity, posing a threat to existing blast-resistant rice cultivars. [32].

By permitting the integration of the desired gene(s) in early breeding generations, marker-assisted selection (MAS) has emerged as a potent method that has advanced the rice breeding effort for blast disease resistance [33]. Many rice cultivars have been improved *via* MAS by pyramiding targeted *R* genes, resulting in the rapid release of rice varieties with durable resistance against blast disease [34]. In recent years, molecular markers have been utilized to capitalize on natural variety and pinpoint the gene of interest influencing essential features in different germplasm [35].

There is indeed a lot of genetic variation in the Indian rice germplasm collection [12,36]. Many of these rice varieties have been reported to have resistance to biotic and abiotic stresses, including blast disease [37-39]. However, the distribution of *R* genes in Indian rice cultivars that confer long-term resistance to leaf and neck blast has not been adequately explored. As a result, it's critical to comprehend *R* gene information in rice germplasm as well as the resistant spectrum of relevant *R* genes against prevailing pathogen races to use the most successful ones in the rice breeding programme to combat blast disease. The present study was carried out to explore the genetic association of 25 mapped resistance genes in 52 rice accessions, including released varieties, advanced breeding materials, and traditional rice varieties using linked/functional markers. The main goal of this study was to find an association between the leaf and neck blast *R* genes, which impart blast resistance to these lines, and novel blast resistance donor sources (*R* genes/alleles).

Materials and Methods

Plant Materials Used in the Current Research

A total of 50 rice accessions were collected based on documented rice blast resistant information from the Rice Genetics laboratory, Crop Improvement Division, ICAR- Vivekananda Parvatiya Krishi Anusandhan Sansthan (VPKAS), Almora, Uttarakhand, India (Tables 1 & 2). The test material includes released varieties (04), advanced breeding materials (44), and traditional rice varieties (02). In addition, two genotypes, PB-1 and Bala, were chosen as leaf and neck blast susceptible controls, respectively (Tables 1 and 2).

Phenotyping of Rice germplasm lines for blast disease resistance

A set of 50 rice hill germplasm collections were evaluated under the natural conditions at the rice blast hotspot area, ICAR-VPKAS, experimental farm, Hawalbagh (29o56'N, 79o40'E, and 1250m MSL), Almora, for their reactivity against leaf and neck blast. The evaluations were carried out in three replications over three years, from 2018 to 2020, during the rainy (Kharif) seasons. Sowings were done in two sets, one for leaf blast evaluations and the other for neck blast evaluations. Each rice entry (30 plants/test entry) was raised in 50 cm long rows on nursery beds with a 10 cm row spacing in a uniform blast nursery for leaf blast (UBN). One line of PB-1 (susceptible check) was sown after every 5 entries of test accessions, as well as along the boundaries, to ensure adequate disease transmission. From 25 days after sowing until the susceptibility check showed 85% of the blast disease symptom, the disease spectrum of all the test entries was recorded. A 0-9 scale devised by IRRI, Philippines [39], was used to visually record the disease reaction on each test entry.

Similarly, the other set was also tested for neck blast disease, but Bala was used as a susceptible control. The severity of the disease was graded on a 0-9 scale (IRRI, 2002), with 0 = no lesion or one or two tiny lesions on the panicles; 1= symptom on several pedicels or secondary branches; 3 = lesions on a few primary branches or the middle part of panicle axis; 5 = moderate infection with lesions covering half of the node or the uppermost internode or the lower part of panicle axis; 7 = heavy infection, lesions abundant on the panicle base or uppermost internode or panicle axis near the base with more than 30% of filled grains; 9 = very heavy infection, around the panicle base or uppermost internode or the panicle axis near the base with less than 30 % of filled grains. At physiological maturity, the disease reaction was recorded, and the affected plants were evaluated on a disease scale, Highly resistant (HR) (0-3 score), moderately resistant (MR) (4-5), and susceptible (S) (6-9) were assigned to the test entries, respectively. Whenever differences in the disease spectrum were recorded, the higher disease was taken into account.

DNA isolation and genotyping

Genomic DNA was extracted from the young leaves of 50 rice germplasm lines and two susceptible controls using the CTAB technique [40]. The quality and quantity of isolated genomic DNA

were determined using a Thermo Fisher Scientific NanoDrop™ 1000 Spectrophotometer. After that, the isolated DNA samples were diluted to a concentration of 25 ng/µl in nuclease-free water for PCR amplification. Molecular profiling of 52 rice lines for the presence of major blast resistance genes was carried out using 25 linked or functional molecular markers. The detailed information on blast resistance genes and their corresponding primer pairs used in this investigation is listed in Table 2. About 25 ng of template DNA, 10 pmol of each forward and reverse primers, 25 mM $MgCl_2$, 2 mM of each dNTPs, 1X Taq buffer, 1U Taq DNA polymerase, and nuclease-free water were used in the PCR amplification. The PCR conditions were set as follows: initial denaturation at 94 °C for 5 minutes was followed by 35 cycles of denaturation for 40 seconds at 94 °C, primer annealing for 40 seconds at varied temperatures (Table 3), and extension for 2 minutes at 72 °C were performed, followed by a final 10-minute extension at 72 °C. To double-check the results, PCR amplification was done twice for each marker. The amplified PCR products were resolved in ethidium bromide-stained 3% agarose gels and the scoring were done for the PCR analysis as presence (1) or absence (0).

Allele scoring and genetic diversity analysis

The presence or absence of an allele was indicated as 1 and 0, respectively, in the amplified PCR products of 25 markers, which were scored as a binary matrix. Using a binary data matrix of 25 markers, the genetic distance and similarity coefficients for 52 rice accessions were calculated. Using the Cervus 3.0 programme (Field Genetics Ltd., London, England) and POPGENE 32 software, different parameters such as the number of different alleles per locus (Na), number of effective alleles per locus (Ne), Shannon's Information Index (I), and Expected Heterozygosity (H_E) for each marker were calculated [41]. Subsequently, a heatmap of all the rice accessions was constructed using the pheatmap package with complete linkage clustering method and euclidean distance measure by R version 4.0.3 statistical software for the presence or absence of 25 markers for both leaf and neck blast.

Association analysis

To study the genetic relationship between blast resistance genes and the disease spectrum, we used TASSEL version 5.0 software with a general linear model (GLM) function [42]. Only the *P*-value was seen in 5% of the permutations for the most significant polymorphism in a region when the GLM model of TASSEL (v 5.0) software was performed with 1000 permutations of data. Using genotypic data collected with 25 molecular markers and pheatmap-based clustering with complete linkage clustering method and Euclidean distance measure, the genetic distance between the 52 rice accessions was estimated using R version 4.0.3 statistical programme.

Population structure analysis

Based on genotyping data from 25 markers, the STRUCTURE software v 2.3.4 [43] was used to evaluate the population structure of 52 rice accessions. Using the admixture and correlated allele

frequencies model, each subpopulation (K) was estimated at different *K* values ranging from one to ten, with five runs per *K* value. A total of 200000 burn-in periods and 200,000 Markov chain Monte Carlo (MCMC) iterations were used in the STRUCTURE runs. Using the STRUCTURE HARVESTER software, the highest delta K (ΔK) value was estimated to determine the most likely *K*-value [44]. The pairwise fixation index (FST) was calculated using principal coordinate analysis (PCoA) based on a binary data matrix of 25 markers, and analysis of molecular variance (AMOVA) was performed using the GenAlEx version 6.502 software [45].

Results

Phenotyping of hill germplasm lines

Initially, the responsiveness of 277 rice accessions to rice blast disease was assessed. From these 277 accessions, we chose 52 genotypes based on their reaction to rice blast disease. i.e., resistant, moderately resistant, and susceptible (Tables 1 & 2).

Table 1 List of 52 rice accession used in this study

Planting materials	Genotypes
Released varieties	VL *Dhan* 158, VL *Dhan* 68, VL *Dhan* 221 and VL *Dhan* 206
Advanced breeding materials	VL 8083, VL 8214, VL 8394, VL 8549, VL 8654, VL 20231, VL 20279, VL 20287, VL 20298, VL 20299, VL 20302, VL 20289, VL 31430, VL 31451, VL 31598, VL 31615, VL 31616, VL 31619, VL 31674, VL 31679, VL 31694, VL 31716, VL 31743, VL 31802, VL 31817, VL 31851, VL 31870, VL 31916, VL 31997, VL 32092, VL 32131, VL 32132, VL 32168, A-57, BL-122, BL-245, GSR-102, GSR-106, GSR-124, GSR-125, GSR-132, GSR-142, VOHP-3102 and VL 32197
Traditional rice varieties	VLK 39 and Someshwar
Susceptible checks	PB-1 and Bala

Table 2 List of rice genotypes along with their pedigree.

Sl. No.	Entry name	Pedigree	Sl. No.	Entry name	Pedigree
1	VL 8083	VL 6394/VL 6446	27	VL 31817	Vivek *Dhan* 82/BL122
2	VL 8214	VL Dhan 81/VR539-2	28	VL 31851	VL 30424/IR78
3	VL 8394	VL6394/VL6446	29	VL 31870	BL 122/IR 785-36
4	VL 8549	VL 3861/VL 6394	30	VL 31916	VL *Dhan* 85/BL 245
5	VL 8654	RCPL 1-45/Vivek Dhan 154	31	VL 31997	Vivek *Dhan* 62/MAS-52
6	VL *Dhan* 158	RCPL 1-45/VL 3861	32	VL 32092	VL *Dhan* 85/VOHP 3102
7	VL 20231	VL *Dhan* 81/Vandana	33	VL 32131	VL 10689/UPRI2005-15
8	VL 20279	VL 20240/Sawdhan	34	VL 32132	VL 10689/UPRI2005-15
9	VL 20287	VHC 1462/VL 10499	35	VL 32168	VL *Dhan* 65/VL30919
10	VL 20298	Annada/C101-A51	36	A-57	-
11	VL 20299	Annada/C101-A51	37	BL-122	-
12	VL 20302	VL *Dhan* 221/ VL 30927	38	BL-245	-
13	VL 20289	VHC 1462/VL 10499	39	VL *Dhan* 221	IR 2053-521-1-1-1/Ch 1039
14	VL 31430	Pant *Dhan* 6/VL 3288	40	VLK 39	China 1039/IR580-19-2-3-1
15	VL 31451	IR 72979/PSB RC 2 (IR 32809-26-3-3)	41	GSR-102	-
16	VL 31598	VL 3861/IR57257-34-1-2-1	42	GSR-106	-
17	VL *Dhan* 68	VL 3861/SR 1818BF-4B-1-2-1-2	43	GSR-124	-
18	VL 31615	VL 3861/SR 1818BF-4B-1-2-1-2	44	GSR-125	-
19	VL 31616	VL 3861/SR 1818BF-4-B1-2-1-2	45	GSR-132	-
20	VL 31619	VL 3861/SR 1818BF-4-B1-2-1-2	46	GSR-142	-
21	VL 31674	C101-A51/*O. minuta*	47	VOHP-3102	Local collection

Sl. No.	Entry name	Pedigree	Sl. No.	Entry name	Pedigree
22	VL 31679	*O. minuta*/Vivek *Dhan* 82	48	VL *Dhan* 206	Pure line selection from Bamni (local variety)
23	VL 31694	Vivek *Dhan* 82/IR57257-34	49	VL 32197	VL *Dhan* 81/Vandana
24	VL 31716	*O. minuta*/IR57257-34	50	Someshwar	Local collection
25	VL 31743	VL 30424/IR32809	51	Bala	N 22/T(N)1
26	VL 31802	VL 66/VL30424	52	PB-1	Pusa 167/Karnal Local

Of 52 rice genotypes, 29 (58%) and 22 (42%) rice genotypes were found to be highly resistant, 18 (36%) and 29 (57%) were moderately resistant, while 05 (6%) and 01 (1%) were highly susceptible to leaf and neck blast, respectively. Incidentally, sixteen genotypes showed high resistance to both leaf and neck blasts (Fig 1).

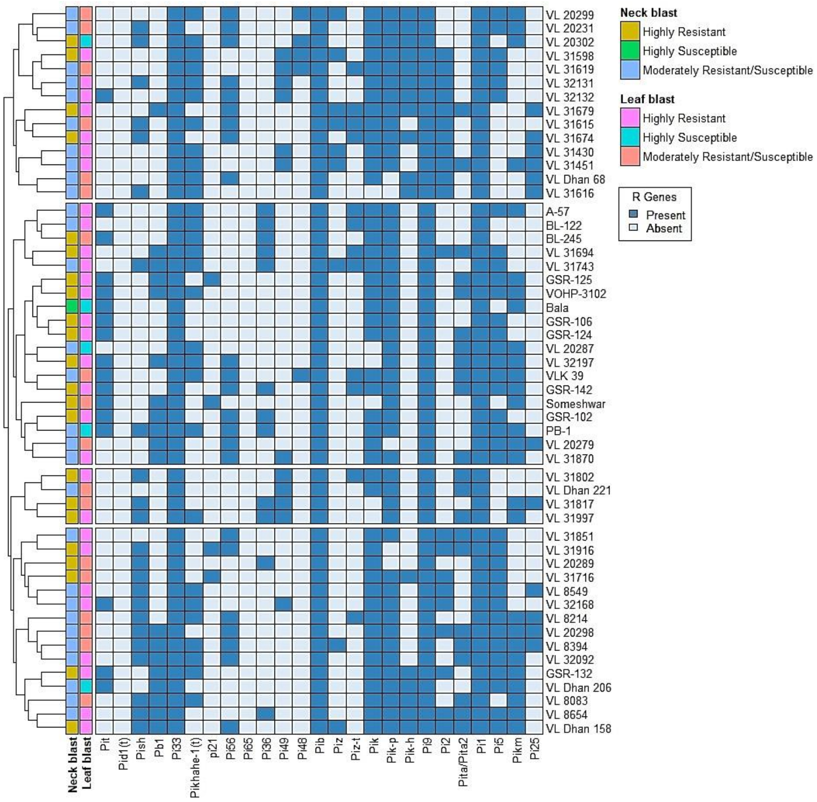

Fig 1. A clustered analysis based on the 25 molecular markers and Heatmap representing the summary of phenotypic and genotypic data of 52 rice genotypes analyzed in this study.

Genetic diversity of blast-resistant R genes

The present study used a set of twenty-five markers (functional/linked markers) that corresponded to the twenty-five *R* genes (Table 3). The gene frequency of the twenty-five blast *R* genes ranged from 32 to 60%, with the number of positive *R*-gene alleles ranging from 0 to 100%. Using a tk59-1 marker to visualize a 733 bp amplicon, the rice blast *R*-gene *Pit* was discovered in 17 rice genotypes. *Pish* on chromosome 1 was amplified with marker RM6648, resulting in a 207-bp band that was detected in 23 genotypes.

Table 3 Details of markers used for molecular screening of blast resistance genes in 52 rice accessions.

Genes	Markers	Forward (5' - 3')	Reverse (5' - 3')	Type of Marker *	Annealing Temperature (°C	References
Pit	tk59-1	ATGATAACCTCATCCTCAATAAGT	GTTGGAGCTACGGTTGTTCAG	FM	54	[48]
Pid1(t)	RM262	CATTCCGTCTCGGCTCAACT	CAGAGCAAGGTGGCTTGC	LM	55	[63]
Pish	RM6648	GATCGATCATGGCCAGAGAG	ACAGCAGGTTGATGAGGACC	LM	55	[34]
Pb1	RM26998	ACGCACGCACATCCTCTTCC	CGGTTCTCCATCTGAAATCCCTAGC	LM	55	[21]
Pi33	RM72	CCGGCGATAAAACAATGAG	GCATCGGTCCTAACTAAGGG	LM	55	[64]
Pikhahe-1(t)	RM17496	TAAACGGTGTGCAGCTTCTG	TATTATGGGCGGTCGCTAAC	LM	54	[65]
pi21	pi21-79-3	GATCCTCATCGTCGACGTCTGGC	AGGGTACGGCACCAGCTTG	InDel	55	[27]
Pi56	CRG4-2	CCTGTCAGTCTTTCCGAGAG	GAATCCGGTAGCTCAAGGTG	Gene-specific	55	[66]
Pi65	SNP_3	TGCCACCAGCCATCTTCAACAT	ACCACATCACTCATCGCCATCC	InDel	54	[71]
Pi36	RM5647	ACTCCGACTGCAGTTTTTGC	AACTTGGTCGTGGACAGTGC	LM	55	[72]
Pi49	RM6094	TGCTTGATCTGTGTTCGTCC	TAGCAGCACCAGCATGAAAG	LM	55	[67]
Pi48	RM5364	GTATTACGCTCGATAGCGGC	GTATCCTTTCTCGCAATCGC	LM	55	[68]
Pib	Pb28	GACTCGGTCGACCAATTCGCC	ATCAGGCCAGGCCAGATTTG	SNP	60	[48]
Piz	Z56592	GGACCCGCGTTTTCCACGTGTAA	AGGAATCTATTGCTAAGCATGAC	SNP	60	[48]
Piz-t	Zt56591	TTGCTGAGCCATTGTTAAACA	ATCTCTTCATATATATGAAGGCCAC	SNP	60	[48]
Pik	K39512	GCCACATCAATGGCTACAACGTT	CCAGAATTTACAGGCTCTGG	SNP	60	[48]
Pik-p	K3957	ATAGTTGAATGTATGGAATGGAAT	CTGCGCCAAGCAATAAAGTC	SNP	60	[48]
Pik-h	Candidate gene marker	CATGAGTTCCATTTACTATTCCTC	ACATTGGTAGTAGTGCAATGTCA	Gene-based marker	55	[69]
Pi9	Pi9-i	GCTGTGCTCCAAATGAGGAT	GCGATCTCACATCCTTTGCT	FNP	54	[52]
Pi2	Pi2-i	CAGCGATGGTATGAGCACAA	CGTTCCTATACTGCCACATCG	FNP	52	[52]
Pita/Pita2	Pita3	AGTCGTGCGATGCGAGGACAGAAAC	GCATTCTCCAACCCTTTTGCATGCAT	SNP	59	[48]
Pi1	RM1233	GTGTAAATCATGGGCACGTG	AGATTGGCTCCTGAAGAAGG	SSR	55	[40]
Pi5	40N23R	TGTGAGGCAACAATGCCTATTGCG	CTATGAGTTCACTATGTGGAGGCT	InDel	55	[40]
Pikm	k2167	CGTGCTGTCGCCTGAATCTG	CACGAACAAGAGTGTGTCGG	InDel	55	[40]
Pi25	CAP1	TGAAATGGGTGAAAGATGAG	GCCACATCATAATTCCTTGA	CAPS	55	[70]

* FM, functional marker; LM, linked marker; InDel, insertion-deletion marker; FNP, functional nucleotide polymorphism; SNP, single nucleotide polymorphism; CAPS, Cleaved Amplified Polymorphism Sequences

A 137-bp amplicon corresponding to the RM26998 marker was used to find the *Pb1* gene on chromosome 11 in 19 genotypes. In 29 rice germplasm lines, the marker RM17496 was able to amplify the *Pikhahe-1(t)* gene with a fragment size of 84 bp. For the recessive blast-resistant gene *pi21*, only four genotypes were determined to be positive. The existence of the blast resistance gene *Pi56* on chromosome 9 was detected using the gene-specific marker CRG4-2, which was found in 23 genotypes. Using the linked marker RM5647, the blast resistance genes *Pi36* (chromosome 8) were discovered in

12 genotypes. The *Pi49* gene, which is located on chromosome 11, was found in 12 genotypes after 182 bp were seen with the RM6094 marker. Using the RM5364 primer, the *Pi48* gene was discovered in five genotypes.

The *R* genes, *Piz*, and *Piz-t* on chromosome 6 were amplified using SNP markers Z56592 and Zt56591, which revealed their presence in nine and twelve entries, respectively. Visualization of 112 bp, 148 bp, and 1500 bp amplicons corresponding to the K39512, K3957, and a gene-based marker, respectively, revealed the *Pik*, *Pik-p*, and *Pik-h* genes on chromosome 11. The genes *Pik*, *Pik-p*, and *Pik-h* were found in 46, 47, and 17 accessions, respectively. The *Pi2* gene was discovered using the Pi2-i primer in twenty-one entries, resulting in positive bands. The major blast resistance gene *Pita/Pita2*, which was scored by visualization of an 861 bp amplicon utilizing the Pita3 marker, was found in 22 genotypes. The *Pi5* gene was discovered in 35 genotypes, which was confirmed using the marker 40N23R. The *Pikm* gene was found in twenty-seven genotypes after PCR amplification. *Pi25* was found in twelve genotypes using the CAP1 primer, which produced a 406-bp amplicon. The R genes *Pi33*, *Pib*, *Pi9*, and *Pi1* were detected in all genotypes; however, the *Pid1(t)* and *Pi65* genes were not discovered in any of the fifty-two genotypes examined in this study. The phenotypic and genotypic data of the 52 rice accessions studied in this investigation are summarized in Figure 1.

Cluster analysis

R-software was used to do the cluster analysis, which separated the 52 rice accessions into two primary clusters. Cluster I had 14 genotypes, seven and four of which were found to be highly resistant to leaf and neck blast, respectively, and three of which, VL31598, VL31679, and VL31674, were shown to be resistant to both leaf and neck blast. Cluster II was divided into three subgroups, the first of which contained a large number of genotypes (19), including nine genotypes resistant to both leaf and neck blast. On the other hand, two susceptible checks (Bala and PB-1) were also clustered together. Subgroup II is made up of four genotypes: VL 31802, VL 31817, VL 31997, and VL *Dhan* 221. Except for VL *Dhan* 221, all three genotypes are resistant to neck blast. Subgroup III has fifteen genotypes, eight and five genotypes showed high resistance to leaf and neck blast, respectively, and three of which were common for both leaf and neck blast resistance, including VL *Dhan* 158, GSR-132, and VL31916. Except for VL *Dhan* 206, the majority of the genotypes exhibited moderate resistance to either leaf or neck blast (Figure 1).

The genotypic data from the 25 markers was used to calculate genetic diversity measures including the number of distinct alleles per locus (Na), the number of effective alleles per locus (Ne), Shannon's Information Index (I), and Expected Heterozygosity (HE). A total of 44 alleles were generated from 25 loci or markers (Table 4). The average number of alleles per locus (Na) was 1.76, with a range of 1 to 2. The number of effective alleles per locus (Ne) ranged from 1 to 1.99, with an average of 1.49. Shannon's Information Index (I) ranged from 0 to 0.692 (Pikm), with an average of

0.42. The Expected Heterozygosity (HE) ranged from 0 (Pid1(t), Pi33, Pi65, Pib, Pi9(Pi9-i), and Pi1) to 0.499 (Pikm) with an average of 0.285.

Table 4 Analysis of the number of alleles, Shannon's Information Index, observed and expected Heterozygosity.

Locus	Na	Ne	I	He
Pit	2.000	1.786	0.632	0.440
Pid1(t)	1.000	1.000	0.000	0.000
Pish	2.000	1.974	0.686	0.493
Pb1	2.000	1.865	0.656	0.464
Pi33	1.000	1.000	0.000	0.000
Pikhahe-1(t)	2.000	1.974	0.686	0.493
pi21	2.000	1.166	0.271	0.142
Pi56	2.000	1.974	0.686	0.493
Pi65	1.000	1.000	0.000	0.000
Pi36	2.000	1.550	0.540	0.355
Pi49	2.000	1.550	0.540	0.355
Pi48	2.000	1.210	0.317	0.174
Pib	1.000	1.000	0.000	0.000
Piz	2.000	1.401	0.461	0.286
Piz-t	2.000	1.550	0.540	0.355
Pik	2.000	1.257	0.358	0.204
Pik-p	2.000	1.210	0.317	0.174
Pik-h	2.000	1.786	0.632	0.440
Pi9 (Pi9-i)	1.000	1.000	0.000	0.000
Pi2 (Pi2-i)	2.000	1.929	0.675	0.482
Pita (Pita3)	2.000	1.954	0.681	0.488
Pi1	1.000	1.000	0.000	0.000
Pi5	2.000	1.786	0.632	0.440
Pikm	2.000	1.997	0.692	0.499
Pi25	2.000	1.550	0.540	0.355

Association analysis

The genetic association of markers with leaf and neck blast disease was examined using the general linear model (GLM) function to see if there was any evidence of a significant link between gene-specific markers and the disease reaction. Only two markers (RM5647 and K39512), which correspond to the blast-resistant genes *Pi36* and *Pik*, respectively, showed a significant association with the neck blast disease, while only three markers (Pi2-i, Pita3, and k2167), which correspond to the blast-resistant genes *Pi2*, *Pita/Pita2*, and *Pikm*, respectively, showed a significant association with the leaf blast disease (Table 5). For leaf blast, the associated markers showed a phenotypic variance of 7.2% to 12.2%. The marker k2167, which is linked to the *Pikm* gene, was shown to have the maximum phenotypic variance. The markers K39512 and RM5647, corresponding to the blast-resistant genes *Pik* and *Pi36*, respectively, showed a phenotypic variance of 4.7 and 5.2% for neck blast. The remaining twenty markers, on the other hand, showed no significant association with blast disease ($p \leq 0.1$).

Table 5 Genetic association of blast resistant genes with rice neck and leaf blast disease in 52 genotypes.

Marker	Neck blast		Leaf blast	
	P value	marker_R^2	*P* value	marker_R^2
Pit	0.31011	0.0206	0.94482	9.68E-05
Pid1(t)	NaN	0	NaN	0
Pish	0.97709	1.67E-05	0.76334	0.00183
Pb1	0.7654	0.0018	0.97428	2.10E-05
Pi33	NaN	0	NaN	0
Pikhahe-1(t)	0.26063	0.02524	0.77591	0.00164
pi21	0.23418	0.02818	0.98122	1.12E-05
Pi56	0.85031	7.19E-04	0.40692	0.0138
Pi65	NaN	0	NaN	0
Pi36	0.1001	**0.05253***	0.46899	0.01054
Pi49	0.74584	0.00212	0.16333	0.03849
Pi48	0.66804	0.00371	0.18689	0.03458
Pib	NaN	0	NaN	0
Piz	0.62943	0.00469	0.31383	0.02028
Piz-t	0.93256	1.45E-04	0.16943	0.03742
Pik	0.10002	**0.0479***	0.1949	0.03337
Pik-p	0.84113	8.11E-04	0.46584	0.01069
Pik-h	0.78264	0.00154	0.90339	2.98E-04
Pi9 (Pi9-i)	NaN	0	NaN	0
Pi2 (Pi2-i)	0.43981	0.01198	0.01374	**0.1154****
Pita (Pita3)	0.62468	0.00482	0.05363	**0.07247****
Pi1	NaN	0	NaN	0
Pi5	0.77264	0.00168	0.33685	0.01846
Pikm	0.1383	0.04341	0.01114	**0.12202****
Pi25	0.64389	0.00431	0.6185	0.005

* & ** Significant at *P* value <0.1 and <0.05 respectively

Population structure analysis

Using STRUCTURE software, all 52 rice genotypes were examined for population structure estimation for leaf and neck blast disease based on 25 markers. The Adhoc Measure *K* peak plateau was discovered to be $K = 2$ (Fig 2), indicating that the complete 52 rice genotypes were divided into two subgroups (SG1 and SG2).

All populations were divided into two major subgroups with eight admixture levels based on an ancestry threshold of >60 % (Table 6). SG1 was made up of the most genotypes identified to be highly resistant to neck blast. The majority of genotypes identified to be highly resistant to leaf blast, on the other hand, were concentrated in SG2. Genotypes with moderate resistance to both leaf and neck blast were clustered together in SG2, while genotypes with high susceptibility to both leaf and neck blast were grouped together in SG1.

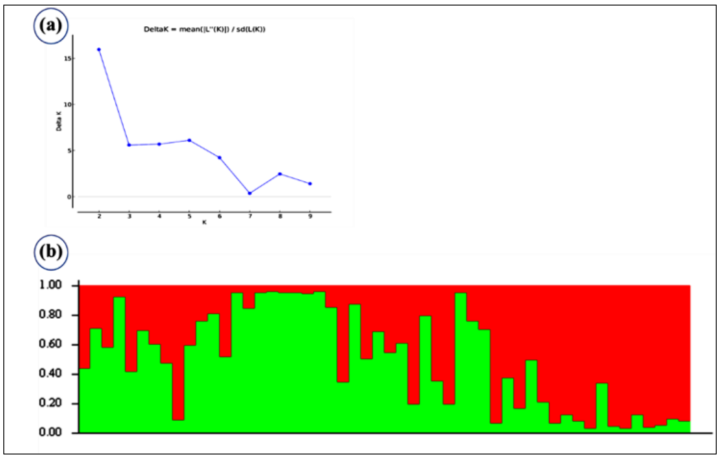

Fig 2. Population structure analysis of 52 rice genotypes (a) The maximum of ad hoc measure *ΔK* was observed to be *K*=3 (b) Estimated population structure graph separated the whole population into two subgroups.

Table 6 Population structure group of 52 genotypes based on inferred ancestry values.

Genotypes	Inferred Ancestry		Structure group
	Q1	Q2	
VL 8083	0.560	0.44	AD
VL 8214	0.291	0.709	SG2
VL 8394	0.419	0.581	AD
VL 8549	0.076	0.924	SG2
VL 8654	0.582	0.418	AD
VL *Dhan* 158	0.302	0.698	SG2
VL 20231	0.398	0.602	SG2
VL 20279	0.527	0.473	AD
VL 20287	0.913	0.087	SG1
VL 20298	0.403	0.601	SG2
VL 20299	0.237	0.763	SG2
VL 20302	0.186	0.814	SG2
VL 20289	0.484	0.516	AD
VL 31430	0.047	0.953	SG2
VL 31451	0.151	0.849	SG2
VL 31598	0.045	0.955	SG2
VL *Dhan* 68	0.039	0.961	SG2
VL 31615	0.048	0.952	SG2
VL 31616	0.05	0.95	SG2
VL 31619	0.055	0.945	SG2
VL 31674	0.04	0.96	SG2
VL 31679	0.145	0.855	SG2
VL 31694	0.657	0.343	SG1
VL 31716	0.123	0.877	SG2
VL 31743	0.495	0.505	AD
VL 31802	0.313	0.687	SG2
VL 31817	0.453	0.547	AD
VL 31851	0.388	0.612	SG2
VL 31870	0.806	0.194	SG1
VL 31916	0.201	0.799	SG2

Genotypes	Inferred Ancestry		Structure group
	Q1	Q2	
VL 31997	0.647	0.353	SG1
VL 32092	0.807	0.193	SG1
VL 32131	0.044	0.956	SG2
VL 32132	0.24	0.76	SG2
VL 32168	0.296	0.704	SG2
A-57	0.933	0.067	SG1
BL-122	0.626	0.374	SG1
BL-245	0.832	0.168	SG1
VL *Dhan* 221	0.503	0.497	AD
VLK 39	0.79	0.21	SG1
GSR-102	0.933	0.067	SG1
GSR-106	0.875	0.125	SG1
GSR-124	0.915	0.085	SG1
GSR-125	0.968	0.032	SG1
GSR-132	0.658	0.342	SG1
GSR-142	0.954	0.046	SG1
VOHP-3102	0.965	0.035	SG1
VL *Dhan* 206	0.878	0.122	SG1
VL 32197	0.964	0.036	SG1
Someshwar	0.944	0.056	SG1
Bala	0.904	0.096	SG1
PB-1	0.92	0.08	SG1

PCoA analysis has been carried out to establish the genetic relationship among the rice genotypes. PCoA analysis revealed that the first two axes explained 17.18 % and 12.29 % of the total variance (Table 7 and Fig 3). In PCoA, leaf blast-resistant genotypes were largely distributed among 1st and 2nd quadrants; on the other hand, most genotypes showed neck blast-resistant were concentrated in the 2nd quadrant. The genotypes found moderately resistant to both leaf and neck blast resistance were mostly distributed among the 1st, 3rd, and 4th quadrants, whereas susceptible genotypes were concentrated in the 2nd quadrant.

Table 7 Percentage of variation explained by the first 3 axes using blast resistance gene in PCoA.

**Axis**	**1**	**2**	**3**
Variation of the individual axis (%)	17.18	12.29	9.21
Cumulative variation (%)	17.18	29.47	38.67

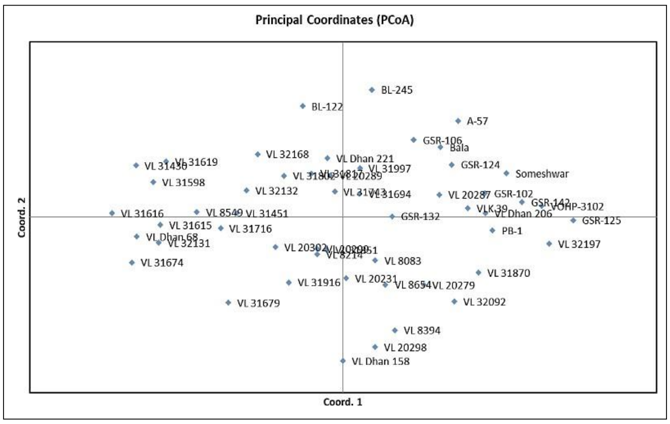

Fig 3. PCoA of 25 molecular markers linked to blast resistance in 52 rice genotypes.

AMOVA analysis

The genetic variations within and between the populations were assessed using AMOVA analysis. The leaf blast score was used to separate 52 rice genotypes into three populations: 29 (HR), 18 (MR), and 05 (S). Similarly, based on neck blast score, 22(HR), 29(MR), and 01(S) were separated. Furthermore, the maximum variance (91%) and (89%) was found within the population, while the least (6%) and (11%), respectively, were found between the populations for leaf and neck blast scores (Figure 4).

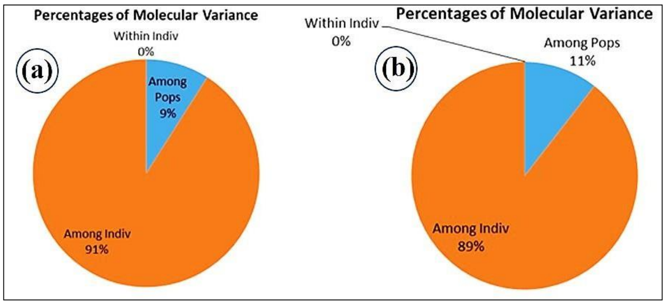

Fig 4 AMOVA analysis based on populations separated with leaf blast scores (a) and neck blast scores (b)

Discussion

Rice genetic diversity has been reduced as a result of large-scale cultivation of high-yielding rice varieties, which have replaced landraces and traditional cultivars, limiting varietal improvement possibilities with existing resources [12,46]. As a result of the widespread cultivation of genetically similar

cultivars across a large area, the pathogen population is subjected to selection pressure, causing it to establish new races. Rice production has become a global threat as a result of the emergence of these new harmful races. However, the problem can be avoided by finding possible donors for unique functional genes or alleles that will help to overcome the disease and ensure future rice harvests [12,37]. The present experiment investigated the genetic diversity of released varieties, advanced breeding materials, and traditional rice varieties for blast resistance genes using 25 molecular markers.

In this study, we used functional/gene-based molecular markers to genotype fifty-two rice hill germplasm collections for 25 major blast-resistant genes, in addition to field evaluations. We examined 52 rice accessions for leaf blast disease resistance in the uniform blast nursery and found that 29 (58%) and 22 (42%) genotypes were highly resistant to leaf and neck blast disease, respectively. Surprisingly, 16 accessions were found to be common for both leaf and neck blast resistance among the highly resistant rice accessions. With one released variety, VL *Dhan* 158, the vast majority of these accessions are advanced breeding materials. ~~Susan et al. [52] examined 288 landraces for rice blast disease resistance and discovered that 75 were highly resistant, 127 were moderately resistant, and 86 were found susceptible. Another study looked at 358 rice accessions for resistance to neck blast and found that 124 cultivars were resistant and 234 cultivars were susceptible, respectively [52].~~

Identification of the individual resistance based on phenotype is typically challenging because it is heavily influenced by developmental stage and environmental factors. However, using a linked marker associated with the *R* genes is the easiest and most reliable way for identifying individual/multiple gene(s) [48]. ~~The identification of blast *R* genes in various germplasms can be done with the use of linked molecular markers [49,50].~~ The frequency of *R*-gene positive alleles ranged from 0% to 100%, with the genetic frequency of 25 major blast resistance genes ranging from 32% to 60%. The most positive alleles for the fifteen resistance genes are found in only two accessions (VL 8394 and VL *Dhan* 158). Our findings are similar to those of Yadav et al. [47] and Susan et al. [52], who reported gene frequencies ranging from 0 % to 100 % in 80 rice varieties released by National Rice Research Institute (NRRI), Cuttack, 9.4 % to 100 % in 32 Chinese rice germplasm, and 6 % to 27 % in 288 Indian landraces, respectively. The *R*-genes *Pib*, *Pi9*, *Pi1*, and *Pi33* appeared to be present in all rice accessions. Our findings match those of Yadav et al. [47], who discovered the *Pib* gene in all eighty rice accessions studied. Similarly, the *Pi9* gene was discovered in 51 Indian landraces [47] and 40 Chinese rice varieties [52]. However, just a few studies have documented the *Pi9* gene's rare prevalence [53,40]. This could be owing to the *Pi9* gene's origin in the wild species *O. minuta* and its subsequent introduction into Indica rice [53]. The *Pi1* gene was detected in 39 landraces with a frequency of 46.98 %, according to Ingole et al. (2014). The presence of the *Pi33* gene was discovered in 77 accessions in another investigation [5].

The genes *Pit*, *Pish*, and *Pikhahe-1(t)* were found in 17, 23, and 29 accessions, respectively. They are also found in the majority of accessions, according to earlier studies [12,47]. In twenty-three acces-

Formatted: Space Before: 6.3 pt

sions, the *Pi56* gene was found. Although it has previously been detected in 27 landraces from north-eastern India [47] and 26 NRRI Cuttack, released varieties, the gene *Pi5* was found in 35 accessions [40]. Nine and twelve accessions, respectively, have the *R* genes *Piz* and *Piz-t*. However, there was no significant correlation was found between these two *R* genes and observed phenotypes. Similarly, they show partial resistance to the genotypes examined by Yadav et al. [47] and Susan et al. [52]. The *Pi2* and *Pita/Pita2* genes were detected in the majority of the rice accessions with high resistance to leaf blast; however, a few genotypes without either of these genes were also resistant to leaf blast and may contain other unique *R*-genes/alleles. Except for VL 20287, which tested positive for the *Pita/Pita2* gene, the genotypes that rated highly vulnerable to leaf blast did not include either of these two genes. Both the *Pi2* and *Pita/Pita2* genes express an NBS-LRR type *R* protein that is resistant to rice leaf blast disease across a broad spectrum of pathogenic races [55,56].

In 19 accessions, the panicle blast resistance gene *Pb1* was found. Only 9 accessions were found to have high resistance to neck blast, while the other ten showed moderate resistance. The *Pb1* gene is a quantitative resistance gene that confers broad-spectrum resistance to all races. Despite having the *Pb1* gene, 10 accessions were found to have only moderate resistance to neck blast. This could be owing to the involvement of at least four QTLs in neck blast resistance, three of which, Chr7, Chr9, and Chr11, have a negative impact on *Pb1*-mediated resistance, while Chr8, on the other hand, has a positive impact. These four QTLs are expected to influence the *Pb1*-mediated resistance either individually or in combination with others [57]. As of today, a few *R* genes, *Pi25*, *Pb1*, *Pi64*, *Pi-jnw1*, and *Pi68(t)* [20-24] and QTLs like, *qNBL-9*, *qNBL-10*, *qNBL-5* [58], *qNB11-1*, *qNB11-3*, *qNB1-1*, *qNB1-2*, *qNB1-3* [59], *qPbh11-1* and *qPbh7-1*, [60] were found to confer resistance to neck blast. Among them, *Pi64*, and *Pi68(t)* were identified for the leaf as well as neck blast resistance. The *pi21* gene was discovered in just four accessions. Surprisingly, all four accessions had high resistance to neck blast, while only the two genotypes had high and moderate resistance to leaf blast disease. The *pi21* gene is a quantitative resistance gene for rice blast disease that offers broad-spectrum resistance [61].

The distance-based clustering was evaluated using genotype data, which divided the 52 germplasm into two primary groupings. Cluster I genotypes was moderately resistant to leaf and neck blast, whereas Cluster II genotypes are highly resistant to both leaf and neck blast. Similarly, the population structure analysis separated the 52 rice accessions into two subpopulations (SG1 and SG2), each with eight admixtures. ~~Surprisingly, the population structure may be able to distinguish between resistant, moderately resistant, and susceptible germplasm. Similarly, the population structure was able to differentiate the 80 NRVs and 288 germplasm into resistant and susceptible [12,47].~~

Formatted: Space Before: 6.3 pt

The leaf and neck blast-resistant genotypes are found in the first and second quadrants of the PCoA analysis, whereas moderately resistant genotypes were found in the first, third, and fourth quadrants. Previous research has also divided resistant and susceptible germplasm into distinct categories [40,47]. A statistical approach for estimating molecular variance in a single species is the analysis of molecular

variance (AMOVA). The AMOVA analysis revealed that there is the highest diversity within the population and minimal diversity between populations.

As a result of association mapping investigations, several genes influencing significant features have been uncovered, and it is now being utilized to deconstruct the genetic basis of many new qualities [62]. Two markers related to blast resistant genes *Pi36* and *Pik* were found to be strongly associated with neck blast resistance, whereas three markers related to blast resistant genes *Pi2*, *Pita/Pita2*, and *Pikm* were found to be significantly associated with leaf blast resistance. Previous research on association mapping and blast disease resistance has shown its effectiveness in identifying markers associated with QTLs and/or resistance genes giving blast resistance [12,33,40]. The identified resistant rice accessions could be used as donors in future breeding projects because they come from a variety of genetic origins. These resistant accessions might then be studied for the existence of novel functional genes/alleles, allowing them to be exploited in rice improvement programs tailored to the needs of agricultural systems.

Conclusions

The identification of resistant germplasm for both leaf and neck blast will be facilitated by phenotyping along with the molecular characterization of blast resistance genes. Our current research on leaf and neck blast screening provided significant germplasm for breeders to employ as parent material for blast resistance transfer, particularly neck blast resistance, in the production of resistant breeding lines. Further identified resistant lines could be a valuable resource for blast resistance gene mapping, particularly in the case of neck blast disease.

Author Contributions: Conceptualization, J.B., R.H., P.S.K., V.H.D., U.N., P.D., M.K.Y., K.K.M., J.P.A. and P.K.B.; methodology, J.B., R.H., P.S.K., V.H.D., U.N., P.D., M.K.Y., K.K.M., J.P.A. and P.K.B.; software, J.B., and A.H.; validation, J.B., R.H., P.S.K., V.H.D., U.N., P.D., M.K.Y., K.K.M., J.P.A. and P.K.B.; formal analysis, J.B. and A.H.; investigation, J.B., R.H., P.S.K., V.H.D., U.N., P.D., M.K.Y., K.K.M., J.P.A. and P.K.B.; data curation, J.B. and A.H.; original draft preparation, J.B., R.H., P.S.K., V.H.D., U.N., P.D., M.K.Y., K.K.M., J.P.A. and P.K.B.; writing-review & editing, A.H. and A.G.; Funding, A.H. and A.G. All authors read, commented on, and approved the final version of the manuscript.

Funding: This research was funded by ICAR-Vivekananda Parvatiya Krishi Anusandhan Sansthan, Almora, Uttarakhand, India. This research was also partially funded by the Taif University Researchers for funding this research with Supporting Project number (TURSP-2020/39), Taif University, Taif, Saudi Arabia.

Institutional Review Board Statement: Not applicable.

Informed Consent Statement: Not applicable.

Data Availability Statement: All data that support the findings of the present study are included in the article/supplementary material

Acknowledgments: We gratefully acknowledge the support of ICAR-Vivekananda Parvatiya Krishi Anusandhan Sansthan, Almora, Uttarakhand, India for financial support and facilities. We also thank Mr. Omkar Pratap, technical officer for his assistance in lab experiments. Also, the authors appreciated Taif University Researchers for funding this research with Supporting Project number (TURSP-2020/39), Taif University, Taif, Saudi Arabia for providing financial support for the publication of this research.

Conflicts of Interest: No conflicts of interest are declared.

References

1. Pennisi, E. Armed and dangerous. *Science* **2010**, 327, 804–805.
2. Fernandez1, J.; Orth, K. Rise of a Cereal Killer: The Biology of *Magnaporthe oryzae* Biotrophic Growth. *Trends Microbiol.* **2018**, 26, 582 – 597.
3. Le, M.T.; Arie, T.; Teraoka, T. Population dynamics and pathogenic races of rice blast fungus, *Magnaporthe oryzae* in the Mekong Delta in Vietnam. *J. Gen. Plant Pathol*. **2010**, 76, 177-182.
4. He, X.; Liu, X.; Wang, L.; Lin, F.; Cheng, Y.; Chen, Z.; Liao, Y.; Pan, Q. Identification of the novel recessive gene *pi55(t)* conferring resistance to *Magnaporthe oryzae*. *Sci. China Life Sci.* **2012**, 55, 141–149.
5. Singh, A.K.; Singh, P.K.; Arya, M.; Singh, N.K.; Singh, U.S. Molecular screening of blast resistance genes in rice using SSR markers. *Plant Pathol. J.* **2015**, 31, 12–24.
6. Ou, S.H. *Rice Diseases*, 2nd ed.; Commonwealth Agricultural Bureaux International: Wallingford, UK, 1985; p. 380.
7. Choi, J.; Park, S.Y.; Kim, B.R.; Roh, J.H.; Oh, I.S.; Han, S.S.; Lea, Y.H. Comparative Analysis of Pathogenicity and Phylogenetic Relationship in *Magnaporthe grisea* Species Complex. *PLoS ONE*, **2013**, 8, 1-8.
8. Jeevan, B.; Rajashekara, H.; Mishra, K.K.; Subbanna, A.R.N.S.; Singh, A.K.; Sharma, D. Finger millet blast disease: Potential threat to global nutrition security. In *Blast Disease of Cereal Crops*. Nayaka S.C., Hosahatti R., Prakash G., Satyavathi C.T., Sharma R.: Nayaka S.C., Hosahatti R., Prakash G., Satyavathi C.T., Sharma R. (eds) Fungal Biology. Springer, Cham. 2021, pp 51–57.
9. Yashaswini, C.; Reddy, N.P.; Pushpavati, B.; Rao, S.C.; Madhav, S.M. Prevalence of Rice blast (*Magnaporthe oryzae*) incidence in South India. Bulletin of Environment, *Pharmacology and Life Sci*. **2017**, 6, 370-373.

10. Ariya-anandech, K.; Chaipanya, C.; Teerasan, W.; Kate-Ngam, S.; Jantasuriyarat, C. Detection and allele identification of rice blast resistance gene, *Pik*, in Thai rice germplasm. *Agric. Nat. Resour*. **2018**, 52, 525-535.
11. Sharma, T.R.; Rai, A.K.; Gupta, S.K.; Vijayan, J.; Devanna, B.N.; Ray, S. Rice blast management through host–plant resistance: retrospect and prospects. *Agric. Res*. **2012**, 1:37–52.
12. Yadav, M.K.; Aravindan, S.; Ngangkham, U.; Raghu, S.; Prabhukarthikeyan, S.R.; Keerthana, U.; Marnd, B.C.; Adak, T.; Munda, S.; Deshmukh, R.; Pramesh, D.; Samantaray, S.; Rath, P.C. Blast resistance in Indian rice landraces: Genetic dissection by gene-specific markers. *PLoS ONE*, **2019**, 14(1), 1-9.
13. Panda, G.; Sahu, C.; Yadav, M.K.; Aravindan, S.; Umakanta, N.; Raghu, S.; Prabhukarthikeyan, S.R.; Lenka, S.; Tiwari, J.K.; Kar, S.; Jena, M. Morphological and molecular characterization of *Magnaporthe oryzae* from Chhattisgarh. *ORYZA-An International Journal on Rice*. **2017**, 54, 330-336.
14. Sahu, C.; Yadav, M.K.; Panda, G.; Aravindan, S.; Umakanta, N.; Raghu, S.; Prabhukarthikeyan, S.R.; Keerthana, U.; Adak, T.; Sharma, V.; Mohanty, M.R. Morphological and molecular characterization of *Magnaporthe oryzae* causing rice blast disease in Odisha. *ORYZA-An International Journal on Rice*. **2018**, 55, 467-472.
15. Jeevan, B.; Gogoi, R.; Sharma, D.; Manjunatha, C.; Rajashekara, H.; Ram, D.; Mishra, K.K.; Mallikarjuna, M.G. Genetic analysis of maydis leaf blight resistance in subtropical maize (*Zea mays* L.) germplasm. *J. Genet*. **2020**, 99, 1-9.
16. Yamaguchi, I. Overview on the chemical control of rice blast disease. Kluwer Academic Publishers. 2004, pp 1-13.
17. Wang, G.L.; Valent, B. Durable resistance to rice blast. *Science*. **2017**, 355, 906–907.
18. Kalia, S.; Rathour, R. Current status on mapping of genes for resistance to leaf- and neck-blast disease in rice. *3 Biotech*, **2019**, 9, 1-14.
19. Ning, X.; Yunyu, W.; Aihong, L. Strategy for use of rice blast resistance genes in rice molecular breeding, *Rice Sci*. **2020**, 27, 263–277
20. Zhuang, J.Y.; Ma, W.B.; Wu, J.L.; Chai, R.Y.; Lu, J.; Fan, Y.Y.; Jin, M.Z.; Leung, H.; Zheng, K.L. Mapping of leaf and neck blast resistance genes with resistance gene analog, RAPD and RFLP in rice. *Euphytica*, **2002**, 128, 363–370.
21. Hayashi, N.; Inoue, H.; Kato, T.; Funao, T.; Shirota, M.; Shimizu, T.; Kanamori, H.; Yamane, H.; Hayano-Saito, Y.; Matsumoto, T.; Yano, M.; Takatsuji, H. Durable panicle blast-resistance gene *Pb1* encodes an atypical CC-NBS-LRR protein and was generated by acquiring a promoter through local genome duplication. *Plant J.* **2010**, 64, 498–510.
22. Ma, J.; Lei, C.; Xu, X.; Hao, K.; Wang, J.; Cheng, Z.; Ma, X.; Ma, J.; Zhou, K.; Zhang, X.; Guo, X.; Wu, F.; Lin, Q.; Wang, C.; Zhai, H.; Wang, H.; Wan, J. *Pi64*, encoding a novel CC-NBS-LRR

protein, confers resistance to leaf and neck blast in rice. *Mol. Plant Microbe Interact*. **2015**, 28, 558–568.

23. Wang, R.; Fang, W.; Guan, C.; He, W.; Bao, Y.; Zhang, H. Characterization and fine mapping of a blast resistant gene *Pi-jnw1* from the japonica rice landrace Jiangnanwan. *PLoS One*, **2016**, 11, 1-12.
24. Devi, S.J.S.R.; Singh, K.; Umakanth, B.; Vishalakshi, B.; Rao, K.V.S.; Suneel, B.; Shusma, S.K.; Kadambari, G.K.M.; Prasad, M.S.; Senguttvel, P.; Shyamaladevi, D.P.; Madhav, M.S. Identification and characterization of a Large Effect QTL from *Oryza glumaepatula* revealed *Pi68(t)* as putative candidate gene for rice blast resistance. *Rice*, **2020**, 13, 1-13.
25. Skamnioti, P.; Gurr, S.J. Against the grain: safeguarding rice from rice blast disease. *Trends Biotechnol.* **2009**, 27:141–150.
26. Liu, J.; Wang, X.; Mitchell, T.; Hu, Y.; Liu, X.; Dai, L.; Wang, G.L. Recent progress and understanding of the molecular mechanisms of the rice- *Magnaporthe oryzae* interaction. *Mol. Plant Pathol*. **2010**, 11:419–427.
27. Fukuoka, S.; Saka, N.; Koga, H.; Ono, K.; Shimizu, T.; Ebana, K.; Hayashi, N.; Takahashi, A.; Hirochika, H.; Okuno, K.; Yano, M. Loss of function of a proline containing protein confers durable disease resistance in rice. *Science*, 2009, 325, 998–1001.
28. Dean, R.A.; Talbot, N.J.; Ebbole, D.J.; Farman, M.L.; Mitchell, T.K.; Orbach, M.J.; Thon, M.; Kulkarni, R.; Xu, J.R.; Pan, H.; Read, N.D.; Lee, Y.H.; Carbone, I.; Brown, D.; Oh, Y.Y.; Donofrio, N.; Jeong, J.S.; Soanes, D.M.; Djonovic, S.; Kolomiets, E.; Rehmeyer, C.; Li, W.; Harding, M.; Kim, S.; Lebrun, M.H.; Bohnert, H.; Coughlan, S.; Butler, J.; Calvo, S.; Ma, L.J.; Nicol, R.; Purcell, S.; Nusbaum, C., Galagan, J.E.; Birren, B.W. The genome sequence of the rice blast fungus *Magnaporthe grisea*. *Nature*. **2005**, 434, 980–986.
29. Kang, S.; Lebrun, M.H.; Farrall, L.; Valent, B. Gain of virulence caused by insertion of a Pot3 transposon in a *Magnaporthe grisea* avirulence gene. *Mol. Plant Microbe Interact*. **2001**, 14, 671-674.
30. Farman, M.L.; Eto, Y.; Nakao, T.; Tosa, Y.; Nakayashiki, H.; Mayama, S.; Leong, S.A. Analysis of the structure of the AVR1-CO39 avirulence locus in virulent rice-infecting isolates of *Magnaporthe grisea*. *Mol. Plant Microbe Interact*, **2002**, 15, 6-16.
31. Bohnert, H.; Fudal, I.; Dioh, W.; Tharreau, D.; Notteghem, J.; Lebrun, M. A putative polyketide synthase/peptide synthetase from *Magnaporthe grisea* signals pathogen attack to resistant rice. *Plant Cell*, **2004**, 16, 2499-2513.
32. Hittalmani, S.; Parco, A.; Mew, T.V.; Zeigler, R.S.; Huang, N. Fine mapping and DNA marker-assisted pyramiding of the three major genes for blast resistance in rice. *Theor. Appl. Genet*. **2000**, 100, 1121–1128.
33. Wang, C.; Yang, Y.; Yuan, X.; Xu, Q.; Feng, Y.; Yu, H.; Wang, Y.; Wei, X. Genome–wide association study of blast resistance in indica rice. *BMC Plant Biol*. **2014**, 14, 1-11.

34. Koide, Y.; Kawasaki, A.; Telebanco-Yanoria, M.J.; Hairmansis, A.; Nguyet, N.T.; Bigirimana, J.; Fujita, D.; Kobayashi, N.; Fukuta, Y. Development of pyramided lines with two resistance genes, *Pish* and *Pib*, for blast disease (*Magnaporthe oryzae* B. Couch) in rice (*Oryza sativa* L.). *Plant Breed*., **2010**, 129, 670–675.
35. Zhang, P.; Liu, X.; Tong, H.; Lu, Y.; Li, J. Association mapping for important agronomic traits in core collection of rice (*Oryza sativa* L.) with SSR markers. *PLoS One*. **2014**, 9, 1-16.
36. Kumbhar, S.D.; Kulwal, P.L.; Patil, J.V.; Sarawate, C.D.; Gaikwad, A.P.; Jadhav, A.S. Genetic diversity and population structure in landraces and improved rice varieties from India. *Rice Sci*. **2015**, 22, 99-107.
37. Vasudevan, K.; Vera Cruz, C.M.; Gruissem, W.; Bhullar, N.K. Large-scale germplasm screening for identification of novel rice blast resistance sources. *Front. Plant Sci*. **2014**, 5, 1-9.
38. Singh, N.; Choudhury, D.R.; Tiwari, G.; Singh, A.K.; Kumar, S.; Srinivasan, K.; Tyagi, R.K.; Sharma, A.D.; Singh, N.K. Singh, R. Genetic diversity trend in Indian rice varieties: an analysis using SSR markers. *BMC Genet*. **2016**, 17, 1-13.
39. International Rice Research Institute (IRRI). Standard Evaluation System for Rice (SES). 2002**.** Available online: http://www.knowledgebank.irri.org/images/docs/rice- standard- evaluation- system.pdf
40. Yadav, M.K.; Aravindan, S.; Umakanta, N.; Shubudhi, H.N.; Bag, M.K.; Adak, T.; Jena, M. Use of molecular markers in identification and characterization of resistance to rice blast in India. *PLoS One*, **2017**, 12, 1–19.
41. Yeh, F.C. Population genetic analysis of co-dominant and dominant marker and quantitative traits. *Belg. J. Bot*. **1997**, 130:129-157.
42. Bradbury, P.J.; Zhang, Z.; Kroon, D.E.; Casstevens, T.M.; Ramdoss, Y.; Bucker, E.S. TASSEL: software for association mapping of complex traits in diverse samples. *Bioinformatics*, **2007**, 23, 2633–2635.
43. Pritchard, J.K.; Stephens, M.; Donnelly, P. Inference of population structure using multilocus genotype data. *Genetics*, **2000**, 155, 945-959.
44. Earl, D.A. STRUCTURE HARVESTER: a website and program for visualizing STRUCTURE output and implementing the Evanno method. *Conservation Genetics Resources*, **2012**, 4, 359–361.
45. Peakall, R.O.; Smouse, P.E. GENALEX 6.5: genetic analysis in EXCEL. Population genetic software for teaching and research. *Bioinformatics*, **2012**, 28, 2537–2539.
46. Tanksley, S.D.; Mc Couch, S.R. Seed banks and molecular maps: unlocking genetic potential from the wild. *Science*, **1997**, 277, 1063–1066.
47. Susan, A.; Yadav, M.K.; Kar, S.; Aravindan, S.; Ngangkham, U.; Raghu, S.; Prabhukarthikeyan, S.R.; Keerthana, U.; Mukherjee, S.C.; Salam, J.L.; Adak, T.; Banerjee, A.; Rath, P.C. Molecular

identification of blast resistance genes in rice landraces from northeastern India. Plant Pathol. **2019**, 68, 537–546.
48. Hayashi, K.; Yoshida, H.; Ashikawa, I. Development of PCR-based allele-specific and InDel marker sets for nine rice blast resistance genes. *Theor. Appl. Genet*. **2006**, 113, 251–260.
49. Jia, Y.; Wang, Z.; Fjellstrom, R.G.; Moldenhauer, K.A.K.; Azam, M.D.A.; Correll, J.; Lee, F.N.; Xia, Y.; Rutger, J.N. Rice *Pi–ta* gene confers resistance to the major pathotypes of the rice blast fungus in the United States. *Phytopathology*, **2004**, 94, 296–301.
50. Ingole, K.D.; Prashanthi, S.K.; Krishnaraj, P.U. Mining for major blast resistance genes in rice landraces of Karnataka. *Indian J Genet Plant Breed*. **2014**, 74, 378–83.
51. Yan, L.; Yan, B.Y.; Peng, Y.L.; Ji, Z.J.; Zeng, Y.X.; Wu, H.L.; Yang, C.D. Molecular screening of blast resistance genes in rice germplasms resistant to *Magnaporthe oryzae*. *Rice Sci*. **2017**, 24, 41–47.
52. Yang, Y.; Zhang, H.; Xuan, N.; Chen, G.; Liu, X.; Yao, F.; Ding, H. Identification of blast resistance genes in 358 rice germplasms (*Oryza sativa* L.) using functional molecular markers. *Eur. J. Plant Pathol*. **2017**, 148, 567–576.
53. Imam, J.; Alam, S.; Mandal, N.P.; Variar, M.; Shukla, P. Molecular screening for identification of blast resistance genes in North East and Eastern Indian rice germplasm (*Oryza sativa* L.) with PCR based makers. *Euphytica*, **2014**, 196, 199–211.
54. Singh, W.; Kapila, R.; Sharma, T.; Rathour, R. Genetic and physical mapping of a new allele of *Pik* locus from japonica rice 'Lijiangxintuanheigu'. *Euphytica*, 205, 889–901.
55. Deng, Y.; Zhu, X.; Shen, Y.; He, Z. Genetic characterization and fine mapping of the blast resistance locus *Pigm(t)* tightly linked to *Pi2* and *Pi9* in a broad-spectrum resistant Chinese variety. *Theor. Appl. Genet*. **2006**, 113, 705–713.
56. Meng, X.; Xiao, G.; Telebanco-Yanoria, M.J.; Siazon, P.M.; Padilla, J.; Opulencia, R.; Bigirimana, J.; Habarugira, G.; Wu, J.; Li, M.; Wang, B.; Lu, G.D.; Zhou, B. The broad-spectrum rice blast resistance (R) gene *Pita2* encodes a novel R protein unique from *Pita*. *Rice*, **2020**, 13, 1-15.
57. Inoue, H.; Nakamura. M.; Mizubayashi, T.; Takahashi, A.; Sugano, S.; Fukuoka, S.; Hayashi, N. Panicle blast 1 (*Pb1*) resistance is dependent on at least four QTLs in the rice genome. *Rice*, **2017**, 10, 1-10.
58. Hittalmani, S.; Srinivasachary, B.P.; Shashidhar, H.E. Identifying major genes and QTLs for field resistance to neck blast in rice. In: Khush GS et al (eds) Advances in rice genetics. Rice Genetics Collection, Los Banos, **2003**, pp 248–250.
59. Noenplab, A.; Vanavichit, A.; Toojinda, T.; Sirithunyad, P.; Tragoonrung, S.; Sriprakhon, S.; Vongsaprom, C. QTL mapping for leaf and neck blast resistance in Khao Dawk Mali 105 and Jao Hom Nin recombinant inbred lines. *Sci. Asia*, **2006**, 32, 133–142.

60. Fang, N.; Wang, R.; He, W.; Yin, C.; Guan, C.; Chen, H.; Huang, J.; Wang, J.; Bao, Y.; Zhang, H. QTL mapping of panicle blast resistance in japonica landrace Heikezijing and its application in rice breeding. *Mol. Breed*. **2016**, 36, 171–179.
61. Angeles-Shim, R.B.; Reyes, V.P.; del Valle, M.M.; Lapis, R.S.; Shim, J.; Sunohara, H.; Jena, K.K.; Ashikari, M.; Doi, K. Marker-assisted introgression of quantitative resistance gene *pi21* confers broad spectrum resistance to rice blast. *Rice Sci*. **2020**, 27, 113–123.
62. Hall, D.; Tegström, C.; Ingvarsson Pär, K. Using association mapping to dissect the genetic basis of complex traits in plants. *Brief. Funct. Genom*. **2010**, 9, 157-165.
63. Chen, X.W.; Li, S.G.; Xu, J.C.; Zhai, W.X.; Ling, Z.Z. Ma, B.T.; Wang, Y.P.; Wang, W.M.; Cao, G.; Ma, Y.Q.; Shang, J.J.; Zhao, X.F.; Zhou, K.D.; Zhu, L.H. Identification of two blast resistance genes in a rice variety, Digu. *J Phytopathol*, **2004**, 152, 77–85.
64. Berruyer, R.; Adreit, H.; Milazzo, J.; Gaillard, S.; Berger, A.; Dioh, W.; Lebrun, M.H.; Tharreau, D. Identification and fine mapping of *Pi33*, the rice resistance gene corresponding to the *Mag-naporthe grisea* avirulence gene ACE1. *Theor. Appl. Genet*. **2003**, 107, 1139–1147.
65. Xu, X.; Chen, H.; Fujimura, T.; Kawasaki, S. Fine mapping of a strong QTL of field resistance against rice blast, *Pikahei– 1(t)*, from upland rice Kahei, utilizing a novel resistance evaluation system in the greenhouse. *Theor. Appl. Genet*. 2008, 117, 997–1008.
66. Liu, Y.; Liu, B.; Zhu, X.; Yang, J.; Bordeos, A.; Wang, G.; Leach, J.E.; Leung, H. Fine–mapping and molecular marker development for *Pi56(t)*, a NBS–LRR gene conferring broad– spectrum re-sistance to *Magnaporthe oryzae* in rice. *Theor. Appl. Genet*. 2013, 126, 985–998.
67. Sun, P.; Liu, J.; Wang, Y.; Jiang, N.; Wang, S.; Dai, Y.; Gao, J.; Li, Z.; Pan, S.; Wang, D.; Li, W.; Liu. X.; Xiao, Y.; Liu, E.; Wang, G.L.; Dai, L. Molecular mapping of the blast resistance gene *Pi49* in the durably resistant rice cultivar Mowanggu. *Euphytica*. **2013**, 192, 45– 54.
68. Huang, H.; Huang, L.; Feng, G.; Wang, S.; Wang, Y.; Liu, J.; Jiang, N.; Yan, W.; Xu, L.; Sun, P.; Li, Z.; Pan, S.; Liu, X.; Xiao, Y.; Liu, E.; Dai, L.; Wang, G.L. Molecular mapping of the new blast resistance genes *Pi47* and *Pi48* in the durably resistant local rice cultivar Xiangzi–3150. *Phyto-pathology*, **2011**, 101, 620–626.
69. Sharma, T.R.; Madhav, M.S.; Singh, B.K.; Shanker, P.; Jana, T.K.; Dalal, V.; Singh, N.K. High-resolution mapping, cloning and molecular characterization of the *Pi-kh* gene of rice, which confers resistance to *Magnaporthe grisea*. *Mol. Genet. Genom*. **2005**, 274, 569–578.
70. Wang, Y.; Wang, D.; Deng, X.; Liu, J.; Sun, P.; Liu, Y.; Hang, H.; Jiang, N.; Kang, H.; Ning, Y.; Wang, Z.; Xiao, Y.; Liu, X.; Liu, E.; Dai, L.; Wang, G.L. Molecular mapping of the blast resistance genes *Pi2–1* and *Pi51(t)* in the durably resistant rice 'Tianjingyeshengdao'. *Phytopathology*, **2012**, 102, 779–786.
71. Zheng, W.; Wang, Y.; Wang, L.; Ma, Z.; Zhao, J.; Wang, P.; Zhang, L.; Liu, Z.; Lu, X. Genetic mapping and molecular marker development for *Pi65(t)*, a novel broad–spectrum resistance gene to rice blast using next-generation sequencing. *Theor. Appl. Genet*. **2016**, 129 1035–1044.

72. Liu, Q.X.; Wang, L.; Chen, S.; Lin, F.; Pan, Q.H. Genetic and physical mapping of *Pi36(t)*, a novel rice blast resistance gene located on rice chromosome 8. *Mol. Genet. Genom*. **2005**, 274, 394–401.
