# Supplementary material for: Phenotypic and Genotypic screening of fifty-two rice (Oryza sativa L.) genotypes for desirable cultivars against blast disease
Source: PLoS One. 2023 Mar 10;18(3):e0280762. doi: 10.1371/journal.pone.0280762 (PMC10004593; doi:10.1371/journal.pone.0280762)
Supplement: Attachment — Submitted filename: 1. Response to Reviewers(1).docx [file pone.0280762.s002.docx]

**Response to reviewers' comments**

We are thankful to both the reviewers and editor for their positive feedback and valuable suggestions to improve our manuscript.

We have addressed all the queries raised by the reviewers. Our responses to the reviewer’s queries are listed below.

**Reviewer #1**

1. The natural screening is ok for testing the field resistance but to know the proper resistant line it is essential to go for artificial screening. Why the authors did not go for that?

**Author's response:** We agree with the reviewer's observation. We used two isolates of *M. oryzae* to conduct artificial screening for leaf blast against entries that demonstrated high resistance in the field conditions and found resistance even at greenhouse experiments in order to confirm the field results. However, till date, full proof artificial screening method for the neck blast disease is not available (therefore, field screening under high disease pressure is more reliable technique so far). The susceptible check in our study recorded the highest disease score, indicating the high disease pressure in the field and the result obtained doesn’t require any further confirmation through artificial screening. Moreover, the field evaluations were conducted at Almora, India, which has been designated as a hotspot location for the rice blast disease. Therefore, the data obtained in the filed screening is reliable and consistent over three years of repeated screening.

2. Neck blast is highly dependent on favorable weather condition coinciding with the flowering time. You have taken different breeding lines which must have different durations so, there is a chance that the lines which are showing resistance may be disease escape.

**Author's response:** Reviewer raised a valid question. Except for a few lines that flowered in 105–107 days, the majority of the rice lines used in this research flowered between 95 and 100 days. The days to flowering were therefore not significantly different across the lines employed in this investigation. In all three years of the experiment (2018 to 2020), the sowing was carried out during the first week of June. Over the course of the study period, we observed maximum mean temperatures of 31 ⁰C and minimum mean temperatures of 19 ⁰C, with an average mean temperature of ~23 ⁰C and a mean relative humidity of >75%, which is extremely favourable for the rice blast disease. Therefore, no question of disease escapes due to weather parameters.

3. Can we report breeding lines which we don't have IET numbers?

**Author's response:** Yes, we are willing to report the breeding lines without an IET number. Since entries participating in AICRP trials are the only ones to which IET numbers can be assigned. Therefore, no issue with reporting without IET numbers.

**Additional Comments from the reviewer:**

1. L.42 - In order to explore the rice blast-resistant sources, we initially performed a large-scale screening of 277 rice accessions → In order to explore the rice blast-resistant sources, initially performed a large-scale screening of 277 rice accessions.

**Authors:** Agreed and included the suggestion

2. L.328 to 331 - Susan et al. [52] examined 288 landraces for rice blast disease resistance and discovered that 75 were highly resistant, 127 were moderately resistant, and 86 were found susceptible. Another study looked at 358 rice accessions for resistance to neck blast and found that 124 cultivars were resistant and 234 cultivars were susceptible, respectively [52]. → delete

**Authors:** Agreed and deleted as per suggestions.

3. L.335 to 336 - The identification of blast R genes in various germplasms can be done with the use of linked molecular markers [49,50]. → delete

**Authors:** Agreed and deleted as per suggestions.

4. L.381 to 383 - Surprisingly, the population structure may be able to distinguish between resistant, moderately resistant, and susceptible germplasm. Similarly, the population structure was able to differentiate the 80 NRVs and 288 germplasm into resistant and susceptible [12,47]. → delete

**Authors:** Agreed and deleted as per suggestions.

=====================================================

**Reviewer #2**

**Authors’ response:** Reviewer-2 comments in the PDF file have been incorporated in the text of the manuscript. Please check all edits in track change mode.
